# Clinical characteristics of antithyroid drug-induced aplastic anemia cases over the past 30 years

**DOI:** 10.3389/fendo.2023.1064723

**Published:** 2023-01-27

**Authors:** Ying Cheng, Xin-Yu Xia, Wei Zhang, Li Ren, Chen-Fu Tian, Dan Liu, Gang Xue

**Affiliations:** ^1^ Department of Endocrinology, The General Hospital of Western Theater Command Chinese People's Liberation Army, Chengdu, Sichuan, China; ^2^ Department of Clinical Medicine, North Sichuan Medical College, Nanchong, Sichuan, China; ^3^ Department of Thyroid and Breast Surgery, The General Hospital of Western Theater Command Chinese People's Liberation Army, Chengdu, Sichuan, China

**Keywords:** antithyroid drug, aplastic anemia, hyperthyroidism, drug adverse reaction, methimazole

## Abstract

**Objective:**

The authors aimed to investigate the clinical characteristics of antithyroid drug-induced aplastic anemia cases over the past 30 years.

**Methods:**

The data of patients with antithyroid drug-induced aplastic anemia were retrieved from PubMed and Wanfang Medical Network databases from 1992 to August 2022. The clinical characteristics, such as age distribution, gender tendency, common symptoms, blood cell count, bone marrow features, treatment strategy, and prognosis, were analyzed.

**Results:**

A total of 17 cases (male:female = 1:16) had been retrieved. Patients’ age ranged from 16 to 74 years (median 50 years). Among them, 82.3% (14/17) of the patients were administered methimazole (MMI), and 78.6% of them had MMI ≥30 mg/day. In addition, 88.2% (15/17) of the patients had sore throat and fever, and 47.1% (8/17) of the patients had hemorrhagic symptoms. Aplastic anemia occurred within 6 months after initiation of the antithyroid therapy in 94.1% of the patients. Agranulocytosis (94.1%) was the most common and earliest blood cell change, and 47.1% of the patients experienced progressive platelet decline during the treatment process. The treatments include timely withdrawal of antithyroid drugs, broad-spectrum antibiotics, granulocyte colony-stimulating factor (G-CSF)/granulocyte-macrophage colony-stimulating factor (GM-CSF), glucocorticoids and other immunosuppressive agents, and supportive treatments such as erythrocyte transfusion and platelet transfusion. Moreover, 70.6% of the patients had complete or near-complete remission within 8 days to 6 weeks.

**Conclusion:**

Aplastic anemia is a rare and serious adverse reaction of antithyroid drugs, which is more common in women. It usually occurs during early treatment with high-dose antithyroid drugs. Most patients have a good prognosis after timely drug ceasing and appropriate treatment.

## 1 Introduction

Antithyroid drugs have been widely used as the first-line treatment for Graves’ disease in the past 70 years. Compared with radioiodine and thyroidectomy, antithyroid drugs do not destroy thyroid tissue and do not result in permanent hypothyroidism, so it is more acceptable to patients. However, antithyroid drugs are associated with multitudinous adverse reactions. Skin rash, urticaria, alopecia, and arthralgia are common minor side effects, whereas agranulocytosis, severe hepatotoxicity, and antineutrophil cytoplasmic antibody-associated vasculitis are rare but serious adverse reactions. These side effects limit the clinical application of antithyroid drugs. Among all of these adverse reactions, hematological abnormalities are rare but important in view of their potentially lethal consequence. A retrospective cohort study had revealed 55 patients with documented hematopoietic damage in 50,385 patients treated with antithyroid drugs (1), including 50 cases of agranulocytosis and five cases of pancytopenia. The antithyroid drug-induced hematopoietic damage includes agranulocytosis, thrombocytopenia, pancytopenia, and aplastic anemia. Agranulocytosis, defined as a granulocyte count below 0.5 × 10^9^/L, is the most common and accounting for 89% of the patients, with an incidence of 0.1%–0.5% ^[2,3]^. The remaining 11% are pancytopenia or aplastic anemia (2, 3), presenting as a decrease in the number of the three types of blood cells with or without bone marrow abnormalities. Previous literature reported that the incidence of aplastic anemia was one-tenth of that of agranulocytosis (4). Therefore, the estimated morbidity of antithyroid drug-induced aplastic anemia was not more than 0.05% despite the lack of relevant investigation.

The pathological mechanism of antithyroid drug-induced hematopoietic damage has not been elucidated; the potential precipitating factors include genetic factors, immune factors, bone marrow suppression, drug toxicity *per sev*, and so on (5–9). Aplastic anemia is a syndrome of hematopoietic failure characterized by a decrease in the hematopoietic tissue of the bone marrow and resulting in a decrease in peripheral blood cells. There are three pathophysiological mechanisms involved in the pathogenesis of aplastic anemia (10): direct marrow damage, constitutional genetic defect, and immune-mediated. These etiological factors contribute to normal bone marrow replaced by non-hematopoietic cells and loss of hematopoietic ability, resulting in a decrease in the number of blood cells. It can be divided into constitutional or acquired. Acquired aplastic anemia is an immune-mediated bone marrow failure syndrome (11). Drugs are a usual etiologic factor inducing acquired aplastic anemia. There is a mass of drugs that are likely to induce aplastic anemia: 1) antineoplastic drugs: temozolomide (12), tyrosine kinase inhibitor (13); 2) antibacterial drugs (14): beta-lactam antibiotics, chloramphenicol, macrolides, trimethoprim/sulfamethoxazole, tetracycline; 3) immunosuppressors: azathioprine (15); 4) non-steroidal anti-inflammatory drugs: diclofenac (16), metamizole sodium (17); 5) others: phenytoin (18), perphenazine (19), and so on. Antithyroid drug-induced aplastic anemia is really rare. Because of the overlap of symptoms and laboratory examinations, it is easily misdiagnosed as agranulocytosis. In addition to the severe infection associated with agranulocytosis, aplastic anemia also manifests severe anemia and bleeding symptoms. Given its lethal consequences, it is worth to pay special attention to.

To have an insight into this rare adverse reaction, we collected relevant case reports over the past 30 years to review and analyze their clinical characteristics, to seek common features, and to provide help for clinical practice.

## 2 Methods

### 2.1 Literature search

Research cases had been screened according to the Preferred Reporting Items for Systematic Reviews and Meta-Analyses (PRISMA) statement. PubMed and Wanfang Medical Network databases were searched for case reports of antithyroid drug-induced aplastic anemia from 1992 to August 2022. “Graves’ disease,” “hyperthyroidism,” “thyrotoxicosis,” “antithyroid drugs,” “methimazole (MMI),” “propylthiouracil (PTU),” “carbimazole (CMZ),” “pancytopenia,” and “aplastic anemia” were used as search keywords. The search language was limited to English and Chinese. The retrieved literature was screened to exclude duplicate data, and the remaining literature was preliminarily screened by title and abstract. The full text of all literature that qualified according to the primary selection criteria was intensively read and further screened according to the inclusion and exclusion criteria.

### 2.2 Study design

The retrieved cases were screened according to the following criteria.

Inclusion criteria: Pancytopenia had occurred after treatment with antithyroid drugs. Aplastic anemia was confirmed by bone marrow aspiration and biopsy, which met the diagnostic criteria of the “Chinese expert consensus on the diagnosis and treatment of aplastic anemia (2017)” (20). The peripheral blood cell test meets at least two of the following three criteria: 1) hemoglobin <100 g/L; 2) blood platelet <50 × 10^9^/L; and 3) absolute neutrophil count <1.5 × 10^9^/L. Bone marrow aspiration showed hypoplasia of the bone marrow, the proportion of non-hematopoietic cells was increased, megakaryocytes were significantly reduced or absent, and erythroid and granuloid cells were significantly reduced. Bone marrow in biopsy was hypoplastic or markedly hypoplastic and showed decreased hematopoietic tissue, increased adipose tissue and non-hematopoietic cells, no increase in reticulin, and no abnormal cells. Other causes were ruled out, and aplastic anemia was confirmed to be induced by the antithyroid drugs.

Exclusion criteria: 1) hematological disorders had been diagnosed previously; 2) aplastic anemia had been diagnosed before antithyroid drug treatment; 3) aplastic anemia due to other hyperthyroidism treatments; 4) lack of bone marrow aspiration, bone marrow biopsy, or other necessary diagnostic data of aplastic anemia; 5) lack of basic clinical information; and 6) aplastic anemia due to other causes.

The clinical data of eligible cases were extracted. The parameters of age, gender, course of hyperthyroidism, duration of medication, peripheral blood cell test, bone marrow examination, and other clinical information were pooled for analysis.

### 2.3 Statistical analysis

Descriptive statistical analysis was adopted. Medians and percentages were calculated by Microsoft Excel software.

## 3 Results

### 3.1 Retrieved Cases

**Figure 1:**
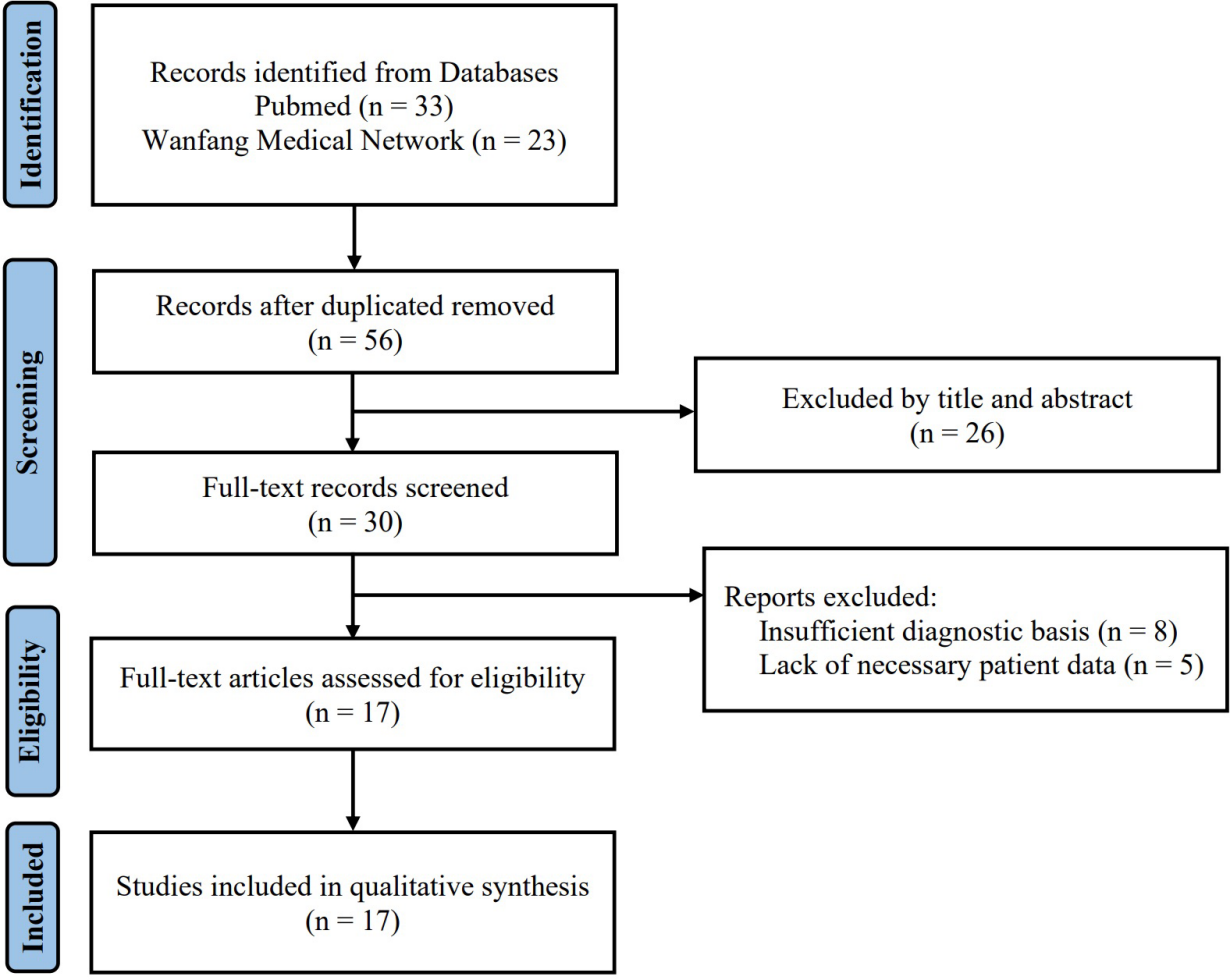
Search strategy and flow of cases.

Using the above search keywords, 56 cases (33 cases from PubMed and 23 cases from Wanfang Medical Network) initially were found after eliminating duplicate cases. Subsequently, 26 cases were excluded by the title and abstract. The full text of the remaining 30 cases was intensively read, and 17 eligible cases (5, 21–35) were finally retrieved according to the inclusion and exclusion criteria (**Figure 1**).

### 3.2 Clinical characteristics of patients

The 17 eligible cases (5, 21–35) were composed of one man and 16 women (male:female = 1:16), aged 16–74 years (median: 50 years). The age distribution is as follows: three cases of ≤20 years, five cases of >20~≤40 years, six cases of >40~≤60 years, and three cases of >60 years. In this study, 64.7% of the patients were young and middle-aged women between 20 and 60 years. The data summary of enrolled patients is shown in **Table 1**.

**Table 1:** Data summary of enrolled patients.

No.	Year	Sex	Age(Y)	Duration of ATD therapy	Symptoms		WBC (×10^9^/L)	N (×10^9^/L)	Hb (g/L)	PLT (×10^9^/L)
					Fever	Hemorrhage				
**1**	1992 (21)	F	17	MMI 45mg/d for 5 weeks	Yes	Yes	1.2	0	90	10
**2**	1996 (22)	F	37	MMI 60mg/d for 8 months	Yes	No	0.5	–	89	48
**3**	1997 (23)	F	51	MMI 60mg/d for 1 month	Yes	No	0.73	0.03	71	15
**4**	1998 (24)	F	58	MMI 30mg/d for 6 months, 17 months of withdrawal, MMI 30mg/d +10mCi ^131^I, 5 months of withdrawal, MMI 30mg/d for 51 days	Yes	No	0.27	0.02	85	41
**5**	2001 (25)	F	16	MMI 30mg/d for 1 month	Yes	Yes	1.7	<0.05	79	12
**6**	2004 (26)	F	53	MMI 30mg/d for 30 days	Yes	No	0.9	<0.09	106	49
**7**	2006 (27)	F	74	MMI 10mg/d for 8 weeks	Yes	Yes	0.3	0.02	79	4
**8**	2007 (28)	F	50	MMI 30mg/d for 50 days	Yes	No	0.7	0.014	114	173
**9**	2008 (29)	F	28	CMZ 30mg/d for 45 days	Yes	No	0.4	0	90	6
**10**	2008 (29)	F	53	MMI 30mg/d for 6 months	Yes	No	0.2	0	90	5
**11**	2008 (30)	F	40	MMI 30mg/d for 1 month	Yes	Yes	0.4	0.064	94	11
**12**	2015 (31)	M	66	MMI 30mg/d for 6 months	No	Yes	1.11	0.22	53	23
**13**	2015 (32)	F	34	CMZ 60mg/d for 6 months	Yes	Yes		0.8	82	40
**14**	2016 (33)	F	16	MMI started with 30mg/d and was gradually reduced to 5mg/d for 4 months	No	Yes	2.0	0.35	106	10
**15**	2018 (34)	F	67	PTU 100mg/d for 20 days	Yes	Yes	0.75	0	100	82
**16**	2019 (5)	F	34	MMI 60mg/d for 28 days	Yes	No	0.58	0.0304	82	24
**17**	2020 (35)	F	59	MMI 20mg/d for 3.5 months	Yes	No	0.64	0.03	78	63

Among them, 14 (82.3%) of 17 patients were prescribed MMI; one patient, PTU; and two patients, CMZ. When patients were diagnosed with aplastic anemia, the drug dosages were separately CMZ 30/60 mg/day, PTU 100 mg/day, and MMI 5–60 mg/day. A total of 78.6% of patients treated with MMI had doses exceeding 30 mg/day. Almost all patients (15/17) had complained of sore throat and fever, and during the fever, the patients’ body temperature was over 39°C. Pharyngitis was the most common clinical manifestation. In addition, some patients had suffered from pulmonary infection, periodontal abscess, perianal infection, and soft tissue infection in other sites. Although most patients had presented severe infection, even sepsis and septic shock, bacterial cultures were often negative. Only two patients were detected with *Escherichia coli* and *Streptococcus pneumoniae*, respectively. Hemorrhagic symptoms were not unusual. Eight patients (47.1%) had various bleeding symptoms, such as epistaxis, purpura and ecchymosis, irregular vaginal bleeding, melena, and hematuria. Three patients died of serious infections, and two of them had accompanying severe gastrointestinal hemorrhage.

Before the diagnosis of aplastic anemia, the duration of antithyroid drug therapy ranged from 20 days to 8 months, with a median of 51 days. Among them, 70.6% of the patients were diagnosed within 3 months of antithyroid drug treatment, and 94.1% of the patients were diagnosed within 6 months. Symptoms appeared earlier; the duration from onset of symptoms to diagnosis of aplastic anemia is generally less than 1 week.

### 3.3 Characteristics of blood cell count and bone marrow

All patients had decreased white blood cell counts, and the vast majority (94.1%) reached to the point of agranulocytosis. The white blood cell count ranged from 0.27 to 2.0 × 10^9^/L, and the granulocyte ranged from 0 to 0.8 × 10^9^/L. Granulocyte count in most patients had reached the lowest point at the beginning of admission and gradually recovered after treatment. The anemia of most patients was mild or moderate. The hemoglobin ranged from 53 to 114 g/L. Hemoglobin was beyond 90 g/L in 40% patients, and only one patient’s hemoglobin had fallen to less than 60 g/L. Nearly all of the patients (16/17) had shown thrombocytopenia, with the lowest blood platelet count ranging from 4 to 173 × 10^9^/L. There were eight patients (47.1%) whose platelet counts were lower than <20 × 10^9^/L. Different from the granulocytes that reached the lowest value on admission, platelet counts in 47.1% of the patients did not reach the bottom at admission, and it further decreased during treatment. Five patients (29.4%) had normal platelet counts on admission and then set to fall dramatically.

Bone marrow aspirations and biopsies of all patients were in accordance with manifestations of aplastic anemia. Bone marrow was hypoplastic or markedly hypoplastic, with decreased hematopoietic cells and increased non-hematopoietic cells, such as lymphocytes, plasma cells, adipocytes, and mast cells. Hematopoietic tissue was replaced by adipose tissue (22, 23, 33). Massive plasma cells infiltrated the bone marrow (25, 26, 30, 32, 34, 36, 37), even the number of plasma cells accounted for 98%, similar to myeloma (25).

### 3.4 Treatment

The overwhelming majority of patients were treated with broad-spectrum antibiotics, and some of them were combined with antiviral and antifungal drugs, except one patient whose principal manifestation was hemorrhage did not receive antibiotics (33). Granulocyte colony-stimulating factor (G-CSF) or granulocyte-macrophage colony-stimulating factor (GM-CSF) were administered in 82.4% of the patients, 64.7% of cases had received glucocorticoids, and cyclosporin was used in 17.6% of the patients. Nearly half (47.1%) of the patients received supportive treatment such as erythrocyte transfusion and platelet transfusion. Human blood immunoglobulin was prescribed to two patients. Due to GM-CSF, blood transfusion and platelet transfusion were ineffective; one patient was prescribed antithymocyte globulin (23).

### 3.5 Prognosis

Most patients (82.4%) had good prognosis with timely withdrawal and appropriate treatment. Among them, 70.6% of the patients completed or nearly completed recovery within 8 days to 6 weeks. There were two patients whose blood cell counts increased slowly and made a complete recovery after 5–6 months. Some patients had been followed up for a long period of time, and no abnormal blood counts had occurred again.

Three patients had exacerbated gradually and eventually died (30, 31, 34). Case 1 (30): A 40-year-old woman. High-dose PTU had been administered due to the symptoms of thyroid crisis. The authors claimed that the cause of death was thyroid crisis induced by severe infection. Case 2 (31): A 66-year-old man. He was the only man among the 17 patients. The cause of death was attributed to gastrointestinal hemorrhage. Case 3 (34): A 67-year-old woman previously diagnosed with metastatic lung adenocarcinoma and myocardial infarction. This patient had died of severe infection and gastrointestinal hemorrhage.

Thyroid function was reported in 9 out of 17 patients after remission of aplastic anemia. Thyroid function of two patients returned to normal, and seven other cases had received further antithyroid treatment. Four patients were treated with radioiodine, and three patients had undergone thyroidectomy.

## 4 Discussion

**Figure 2:**
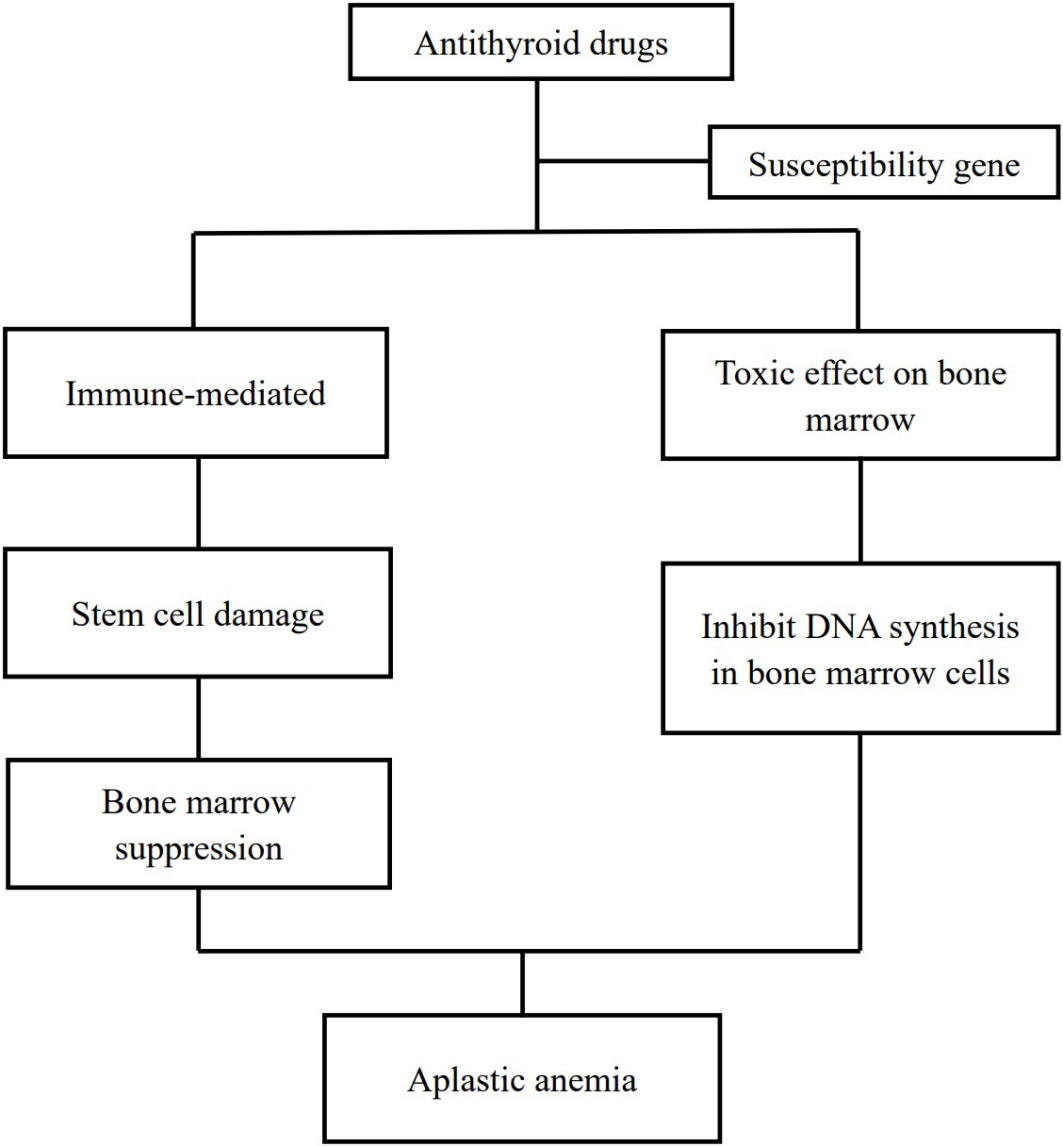
Mechanism of antithyroid drugs induced aplastic anemia.

Aplastic anemia is a rare adverse effect of antithyroid drugs. Up to now, there are less than 100 cases of antithyroid drug-induced aplastic anemia that had been reported. The pathogenesis of aplastic anemia due to antithyroid drugs is an enigma. It is hypothesized that two pathways contribute to antithyroid drug-induced aplastic anemia with the involvement of some susceptibility genes (24, 38): drug-induced immune responses damage bone marrow stem cells and lead to myelosuppression (9, 25, 26, 29, 39) and direct toxic effects of antithyroid drugs on the bone marrow (40, 41) (**Figure 2**).

We have retrieved relevant case reports in PubMed and Wanfang Medical Network databases over the past 30 years and extracted 17 eligible cases. This is the first investigation covering a Chinese database. The cases of antithyroid drug-induced aplastic anemia are mainly concentrated in women aged 20–60 years, which is consistent with the predisposed population of Graves’ disease. Unlike agranulocytosis that is at higher risk in older patients, aplastic anemia does not present a prominent age predisposition. Both have a similar gender tendency and tend to occur in women. The male-to-female ratio of agranulocytosis was 1:7–10.4 (42, 43), and it was as high as 1:16 in the aplastic anemia patients. It remains to be confirmed whether the prominent gender tendency was caused by influencing factors such as sex hormones.

Almost all antithyroid drug-induced aplastic anemia (94.1%) had occurred within 6 months after treatment, and most (70.6%) occurred within 3 months. The occurrence time was close to that of agranulocytosis (7, 44). Antithyroid drug-induced aplastic anemia may also occur after repeated drug exposures (24, 40, 45). The case reported by Mezquita et al. (24) developed aplastic anemia after the third exposure to MMI. Previous literature reported a case that had the onset of aplastic anemia even after the fourth exposure to antithyroid drugs (45).

Unlike some patients with agranulocytosis, who lack clinical symptoms and were found during routine blood examination (2, 43), patients with aplastic anemia all manifested a variety of symptoms. The most common symptoms are sore throat and pyrexia. Decreased white blood cells and granulocytes are the earliest and most common abnormalities of peripheral blood examination. Therefore, aplastic anemia is easy to be misdiagnosed as agranulocytosis in the early stage of the disease. We hypothesized that agranulocytosis and aplastic anemia might share a similar pathogenesis, and they are the different stages of the disease. In the early stage, the antithyroid drugs induce granulocytopenia, but there is no significant reduction in bone marrow hematopoietic tissue, and these patients might have a good response to G-CSF treatment. As the disease progresses, the bone marrow is further suppressed and granulocytes decreased gradually, followed by a decrease in megakaryocyte and erythroid hypoplasia. These patients may be refractory to treatment with G-CSF. Another prominent symptom was hemorrhage, which would be insidious or very serious. It includes irregular vaginal bleeding, skin petechiae and ecchymosis, or severe gastrointestinal hemorrhage. Intracranial hemorrhage had also been reported in previous literature (45, 46). Hemorrhage was associated with a decrease in platelet count. Many patients had normal platelet counts at the earlier stage. Nearly half of the patients experienced a dramatic decrease in platelet count during treatment. There was one patient whose platelet count decreased abruptly from 233 to 10 × 10^9^/L within 5 days (21). Anemia was often not serious in most cases. Palpitations and shortness of breath are easily mistaken for hyperthyroidism symptoms; hence, anemia is ignored frequently unless there is significant pallor of the skin and mucous membranes. The duration of antithyroid drug treatment ranged from 20 days to 8 months before the diagnosis of aplastic anemia, and the onset of symptoms was earlier. In previous reports, some patients complained of sore throat and fever within a few days of antithyroid drug treatment (39). Meanwhile, we found that the severity of symptoms was not in accordance with blood cell counts.

A total of 14 out of the 17 cases were induced by MMI, which may have something to do with the fact that MMI is the most used antithyroid drug. High doses of MMI are believed to be associated with the induction of aplastic anemia, whereas the dose relation of PTU is not clear. Nearly 80% of the patients had a dose of MMI exceeding 30 mg/day at the time of aplastic anemia diagnosis. However, the relationship between antithyroid drug dosage and hematological adverse reactions is still controversial because high-dose MMI was often used in the early stage of hyperthyroidism treatment, which is also the time of drug-induced immune activation.

The patient’s bone marrow had dysplasia; hematopoietic cells decreased and were replaced by adipose tissue. The adipose in bone marrow could inhibit hematopoietic cells through various pathways (47). Some patients’ bone marrow was infiltrated by massive plasma cells, even presenting like myeloma (25). It indicated that humoral immunity was involved in the pathogenesis of antithyroid drug-induced aplastic anemia (48). However, no myelosuppression-related antibodies had been detected, and not all patients have increased plasma cells in the bone marrow. Therefore, it is speculated that there are also other mechanisms involved in the process of antithyroid drug-induced aplastic anemia.

The key to treat antithyroid drug-induced aplastic anemia is early diagnosis and timely drug withdrawal. Since almost all patients presented with infection symptoms and agranulocytosis, the administration of broad-spectrum antibiotics is essential, although it is difficult to identify the pathogenic bacteria. The therapeutic effect of G-CSF/GM-CSF has not been unanimously affirmed (23, 26, 29, 34). Most patients have no severe anemic symptoms and the hemoglobin was not very low, but in fact, erythrocyte transfusion was also a necessary supportive treatment due to serious hemorrhage. Thrombocytopenia is an important influencing factor of unfavorable prognosis, and platelet transfusion is a very effective treatment measure. In previous literature, two patients died of intracerebral hemorrhage when platelet transfusion was not available (45, 46). Eltrombopag, a thrombopoietin receptor agonist, has also been proven to be effective. The use of glucocorticoids has always been controversial, and although most patients are treated with glucocorticoids, about half of them were ineffective (5, 20, 29, 30, 32, 34). Glucocorticoids should be prescribed carefully in view of the risk of infection exacerbation and hemorrhage. Other treatment measures include cyclosporin, antithymocyte globulin, anabolic hormones, erythropoietin, and so on. Most patients had been in remission after the above treatment. There is no case report on bone marrow transplantation, but it should still be considered when other treatments have failed.

Antithyroid drug-induced agranulocytosis generally recovers within 2 weeks after drug withdrawal (43), while recovery from aplastic anemia takes longer, mostly within 6 weeks. Although most cases had a good prognosis, there were still three deaths, which were mainly related to old age (31, 34), underlying diseases (34), hemorrhage (31), failure to withdraw from antithyroid drugs in time (30), and other factors. After the remission of aplastic anemia, radioiodine (5, 24–26) or thyroidectomy (29, 32) was performed if the hyperthyroidism had not been controlled. There is no evidence to indicate which therapeutic strategy is more advantageous.

Graves’ disease is a complicated autoimmune disease, and it *per se* can cause some abnormalities such as abnormal liver function (49) or hematologic damages (8, 50–53); therapeutic measures such as antithyroid drugs and radioiodine may lead to similar manifestations through different mechanisms. In the process of literature retrieval, we also found aplastic anemia induced by radioiodine treatment (54). Regardless of treating with antithyroid drugs, radioiodine, or thyroidectomy, blood cell counts could return to normal accompanied by the improvement of thyroid function (50–52), which implied that the occurrence of aplastic anemia might be related to the change of thyroid function and the decrease in thyroid hormone.

Through the review of previous reported cases, we summarized some common clinical characteristics of patients with antithyroid drug-induced aplastic anemia, such as it is more common in women, mostly in the early 6 months after drug administration, and the onset time of agranulocytosis was earlier than that of thrombocytopenia. Unfortunately, there are no clinical or biochemical indexes that have been found to predict the onset of such adverse reactions. Meanwhile, we should be aware that case reports are of limited scientific value. Further research is needed to verify the results of this systematic review.

## Data availability statement

The original contributions presented in the study are included in the article/supplementary material. Further inquiries can be directed to the corresponding author.

## Author contributions

YC and GX takes responsibility for the design of study and the integrity of the data. X-YX, C-FT, and DL were involved in data collection. WZ and LR did the literature review. YC and X-YX were responsible for data analysis. YC drafted the manuscript and GX revised it. All authors contributed to the article and approved the submitted version.
